# Global, regional and national burdens of Nasopharynx cancer in the adolescents and young adults from 1990 to 2019 and its predictions

**DOI:** 10.1186/s12885-024-12480-7

**Published:** 2024-06-11

**Authors:** Guoxin Huang, Yaojie Wang, Bingqing Qin, Jing Zeng, Huazhang tan, Dongdong Zhang, Qiuyang Wang

**Affiliations:** 1 Department of Otolaryngology, Xiangyang No.1 People’s Hospital, Hubei University of Medicine, 15 Jiefang Road, Fancheng District, Xiangyang, 441000 Hubei China; 2 Department of Evidence-Based Medicine Center, Xiangyang No.1 People’s Hospital, Hubei University of Medicine, Xiangyang, 441000 China; 3 Center Laboratory for Translational Medicine, Xiangyang No.1 People’s Hospital, Hubei University of Medicine, Xiangyang, China; 4 Department of Oncology, Xiangyang No.1 People’s Hospital, Hubei University of Medicine, Xiangyang, Hubei China; 5 Department of Paediatrician, Xiangyang No.1 People’s Hospital, Hubei University of Medicine, Xiangyang, China

**Keywords:** Nasopharynx cancer, Adolescents and young adults, Disease burden, Prediction

## Abstract

**Purpose:**

To use data from the Global Burden of Disease (GBD) Study 2019 to report the global, regional and national rates and trends of deaths incidence, prevalence, disability-adjusted life years (DALYs) for Nasopharynx cancer (NPC) in adolescents and young adults (AYAs).

**Methods:**

Data from the GBD 2019 were used to analyze deaths incidence, prevalence and DALYs due to NPC at global, regional, and national levels. Joinpoint regression analysis was used to calculate the average annual percentage changes (AAPC). The association between incidence, prevalence and DALYs and socioeconomic development was analyzed using the GBD Socio-demographic Index (SDI). Finally, projections were made until 2030 and calculated in Nordpred.

**Results:**

The incidence, prevalence, death and DALYs rates (95%UI) due to NPC 0.96 (0.85–1.09, 6.31 (5.54–7.20),0.20 (0.19–0.22), and 12.23(11.27–13.29) in 2019, respectively. From 1990 to 2019, the incidence and prevalence rates increased by 1.79 (95% CI 1.03 to 2.55) and 2.97(95% CI 2.13 to 3.82) respectively while the deaths and DALYs rates declined by 1.64(95%CI 1.78 to 1.49) and 1.6(95%CI 1.75 to 1.4) respectively. Deaths and DALYs rates in South Asia, East Asia, North Africa and Middle East decreased with SDI. Incidence and prevalence rates in East Asia increased with SDI. At the national level, the incidence and prevalence rates are high in China, Taiwan(China), Singapore, Malaysia, Brunel Darussalam, Algeria, Tunisia, Libya and Malta. Meanwhile, the deaths and DALYs rates are still high in Malaysia, Brunel Darussalam, Greenland and Taiwan(Province of China). The deaths and DALYs rates are low in Honduras, Finland and Norway. From the 2020 to 2030, ASIR、ASPR and ASDR in most regions are predicted to stable, but DALYs tends to decline.

**Conclusion:**

NPC in AYAs is a significant global public problem. The incidence, prevalence, and DALYs rates vary widely by region and country. Therefore different regions and countries should be targeted to improve the disease burden of NPC.

## Introduction

Nasopharyngeal carcinoma (NPC) is an epithelial carcinoma originating from the mucosal lining of the nasopharynx, where tumours are frequently observed in the pharyngeal crypts. Despite originating from similar cellular or tissue lineages, NPC and other epithelial head and neck tumours are distinctly different. In 2018, there were an estimated 129,079 confirmed cases of NPC internationally, with Asia accounting for about 85 percent of these cases. In total, an estimated 72,987 people died from NPC, making it the 23rd most common cancer in the world and the 21st most common cause of cancer death globally [1]. For decades, NPC have been prevalent in someregions and countries, including East and Southeast Asia, the Arctic, the indigenous populations of North Africa and the Middle East, and Southern China [2]. A subgroup of patients with NPC globally are those in the transition phase between childhood and old age, that is, those aged 15–39 years, which can be referred to as adolescents and young adults. This subgroup will experience dramatic physical, emotional and psychosocial changes and important life events, a characteristic period of life. Previously Bray et al. found that in low-risk populations, NPC incidence peaks moderately in young adulthood (age 15–19 years), stabilises or declines slightly by age 35–39 years, and then rises to a second, higher peak at approximately age 65–79 years [3]. However, there is insufficient research on how the burden of disease among adolescents and young adults with NPC varies across countries, regions, and ethnicities, so it is critical to focus on the prevention, diagnosis, and treatment of NPC disease in this vulnerable group.So the article focus on this problem to carry out research.

## Methods

### Overview

The GBD 2019 offers extensive data on Nasopharyngeal Cancer (NPC) at the global, regional, and national levels. Adolescents and Young Adults (AYAs), defined here as those aged between 15 and 39 years from 1990 to 2019, serve as the focus for this examination. The study evaluates the NPC burden by analyzing metrics such as cases of death, death rates, instances of incidence, incidence rates, prevalence, prevalence rates, Disability-Adjusted Life Years (DALYs), and DALY rates.

### Data sources

The data for the study was sourced via the Global Health Data Exchange's search function, setting specific parameters that include the "GBD Estimate" to identify causes of death or injury, and measures like "Deaths, Incidence, Prevalence, and DALYs". These settings focused on "Nasopharynx cancer" across various locations globally and by Socio-demographic Index (SDI) categories. Parameters also specified age (15–39 years), gender (both sexes), and encompassed all years to ensure a thorough analysis.

### Estimation framework

The Global Burden of Disease (GBD) 2019 framework calculates health metrics using detailed methodologies previously discussed [4–6]. The methodology from GBD 2019 involves calculating prevalence and incidence rates per 100,000 by dividing total and new case numbers by the population size, respectively. DALYs are used to measure the combined years lost due to disability and premature death, providing an aggregate indication of health loss attributed to disease from its beginning until death. This approach meticulously quantifies the burden of disease, considering both longstanding and recent diagnoses within the population.

### Socio-demographic index

The Socio-demographic Index (SDI) serves as a combined metric reflecting a country or region's developmental level, integrating per capita income, average education duration, and fertility rates into a singular score ranging from 0 to 1. Higher scores denote more advanced development. Globally, 204 regions or countries are evaluated, classified into five SDI categories from low to high, facilitating comparisons and analyses within the academic community.

### Statistical analysis

The statistical analysis utilized R software version 4.2.2, analyzing global to national data from 1990 to 2019, focusing on age-standardized incidence, prevalence, death rates, and DALYs. Uncertainty was quantified with 95% confidence intervals. EAPC values are utilized to track trends in age-standardized rates, indicating increases when both EAPC and the lower 95% confidence interval are positive. Smoothing splines models assess the relationship between the burden of NPC across various metrics and the Socio-demographic Index (SDI) in 21 regions. Decomposition analysis explores the roles of population aging, growth, and epidemiological shifts in affecting NPC burden from 1990 to 2019, providing insights into the complex dynamics influencing these health outcomes [7]. Frontier analysis is utilized to identify potential health improvements achievable within the current developmental level across 204 countries. This technique generates a nonlinear frontier, representing the minimum expected disease burden given a country's development. The gap between a nation's current age-standardized DALYs and this frontier highlights the possible health benefits not yet realized, offering insights into where and how interventions could effectively reduce the disease burden based on development [8]. The Nordpred method, widely recognized in scholarly discussions, is applied across Socio-demographic Index (SDI) regions and in 21 regional forecasting models globally. It employs age-period-cohort analysis to predict future disease trends, providing a structured framework for estimating future health scenarios based on historical data and demographic dynamics [9, 10].

## Results

### The incidence, prevalence, deaths, DALYs of Nasopharynx cancer at global level

From 1990 to 2019, while the incidence and prevalence of NPC in AYA population has increased, the number of deaths, death rates, DALY and DALY rates has declined.Details are in Table 1.

**Table 1:** Global Age-standardized Incidence, Age-standardized Prevalence, Age-standardized DALYs, and estimated annual percentage change of nasopharyngeal carcinoma from 1990 to 2019

1990								
	Incidence		Prevalence		Deaths		DALYs	
Location	Cases(95%UI)	Rate per 100,000 people(95%UI)	Cases(95%UI)	Rate per 100,000 people(95%UI)	Cases(95%UI)	Rate per 100,000 people(95%UI)	Cases(95%UI)	Rate per 100,000 people(95%UI)
Global	12579.17(11154.91–13857.82)	0.57(0.50–0.63)	59273.84(52656.24–65622.87)	2.70(2.40–2.99)	7250.28(6433.79–8015.38)	0.33(0.29–0.36)	427490.38(379790.26–472411.73)	19.48(17.31–21.53)
Oceania	5.21(3.60–7.31)	0.19(0.13–0.27)	19.21(13.51–26.13)	0.72(0.51–0.99)	3.97(2.70–5.61)	0.15(0.10–0.21)	228.17(156.54–321.22)	8.66(5.94–12.20)
High-income North America	395.33(365.67–426.45)	0.34(0.32–0.37)	2647.27(2427.37–2878.93)	2.34(2.14–2.54)	73.95(71.23–76.90)	0.06(0.06–0.07)	4493.40(4321.45–4681.22)	3.97(3.82–4.13)
Tropical Latin America	50.47(47.13–53.95)	0.07(0.07–0.08)	210.02(194.19–225.92)	0.32(0.30–0.35)	34.49(32.34–36.71)	0.05(0.05–0.06)	2095.16(1962.05–2228.42)	3.25(3.05–3.46)
East Asia	7208.41(6127.31–8393.20)	1.27(1.08–1.48)	34256.85(28804.70–40466.35)	6.04(5.08–7.13)	4111.97(3506.74–4789.65)	0.72(0.61–0.84)	241577.39(206143.26–281107.20)	42.60(36.35–49.57)
Australasia	32.77(25.46–39.96)	0.40(0.31–0.49)	207.20(154.83–257.32)	2.54(1.89–3.15)	7.98(7.01–9.06)	0.09(0.08–0.11)	475.20(419.54–538.31)	5.82(5.14–6.60)
Low-middle SDI	1625.14(1356.27–1907.73)	0.36(0.30–0.42)	6009.82(5004.47–7067.59)	1.34(1.12–1.58)	1255.25(1051.96–1488.44)	0.28(0.23–0.33)	74159.65(61924.58–87923.24)	16.65(13.90–19.74)
Eastern Europe	136.99(120.06–157.40)	0.15(0.13–0.18)	708.28(598.95–836.08)	0.82(0.69–0.97)	65.94(60.52–72.50)	0.07(0.07–0.08)	3934.05(3622.51–4313.31)	4.58(4.22–5.02)
High SDI	1643.10(1543.78–1749.81)	0.50(0.47–0.54)	10508.72(9788.52–11304.34)	3.25(3.02–3.49)	408.95(392.99–426.49)	0.12(0.12–0.13)	24177.09(23175.54–25186.21)	7.48(7.17–7.79)
Caribbean	21.23(17.02–25.20)	0.14(0.11–0.16)	100.59(81.01–123.16)	0.67(0.54–0.83)	12.19(9.62–14.55)	0.08(0.06–0.09)	749.44(590.24–896.53)	5.05(3.97–6.04)
Middle SDI	4999.07(4389.87–5567.26)	0.66(0.58–0.74)	21404.86(18647.28–23922.76)	2.86(2.49–3.20)	3302.46(2940.80–3689.42)	0.44(0.39–0.49)	194536.32(172783.74–217381.49)	26.056(23.14–29.11)
Western Sub-Saharan Africa	80.63(61.62–103.24)	0.11(0.08–0.14)	284.02(217.49–363.82)	0.39(0.30–0.51)	64.17(49.407–83.07)	0.09(0.06–0.11)	3745.08(2886.54–4837.62)	5.26(4.05–6.80)
Central Europe	72.36(66.47–79.04)	0.15(0.14–0.17)	340.25(305.74–379.01)	0.73(0.66–0.82)	41.98(39.20–45.15)	0.09(0.08–0.09)	2437.54(2275.84–2616.29)	5.29(4.94–5.68)
Central Latin America	43.14(40.76–45.88)	0.06(0.05–0.06)	184.73(172.32–198.94)	0.27(0.25–0.29)	28.52(27.08–30.09)	0.04(0.03–0.04)	1743.69(1658.56–1839.80)	2.55(2.43–2.69)
Southeast Asia	1215.67(998.33–1401.92)	0.61(0.50–0.71)	4710.58(3867.27–5423.94)	2.39(1.96–2.75)	895.36(739.88–1027.39)	0.45(0.37–0.52)	52789.24(43537.84–60767.37)	26.82(22.12–30.87)
Central Sub-Saharan Africa	24.15(16.99–32.40)	0.11(0.08–0.15)	88.00(62.14–118.19)	0.42(0.29–0.56)	18.28(13.23–24.28)	0.08(0.06–0.11)	1111.53(800.85–1481.86)	5.35(3.85–7.13)
Andean Latin America	7.62(6.57–8.79)	0.04(0.04–0.05)	29.44(25.21–34.28)	0.19(0.16–0.22)	5.64(4.87–6.50)	0.03(0.03–0.04)	343.48(295.87–395.55)	2.22(1.91–2.55)
Low SDI	526.78(406.25–668.09)	0.27(0.20–0.34)	1853.78(1430.4–2343.17)	0.95(0.73–1.20)	416.64(319.57–528.32)	0.21(0.16–0.27)	24502.74(18808.37–31109.65)	12.64(9.70–16.05)
Western Europe	677.26(602.58–757.35)	0.47(0.41–0.52)	4565.48(4014.64–5162.47)	3.16(2.78–3.58)	122.13(116.24–128.07)	0.08(0.08–0.09)	7487.55(7103.72–7842.56)	5.19(4.93–5.44)
High-middle SDI	3781.34(3167.22–4606.68)	0.78(0.65–0.95)	19479.67(15921.90–24205.95)	4.02(3.29–5.01)	1864.71(1575.51–2212.18)	0.38(0.32–0.45)	109980.77(93060.81–129972.10)	22.74(19.24–26.88)
North Africa and Middle East	532.03(435.82–628.05)	0.39(0.32–0.46)	2649.85(2127.45–3165.73)	1.95(1.56–2.33)	279.84(231.68–330.65)	0.20(0.17–0.24)	2649.85(2127.45–3165.73)	1.95(1.56–2.33)
Central Asia	41.03(37.39–45.58)	0.14(0.13–0.16)	169.85(154.22–190.06)	0.59(0.54–0.66)	28.39(25.91–31.74)	0.09(0.09–0.11)	1754.88(1604.15–1955.61)	6.16(5.63–6.86)
South Asia	1465.94(1250.65–1727.38)	0.33(0.28–0.39)	5251.09(4480.12–6199.62)	1.21(1.03–1.43)	1169.77(985.13–1381.84)	0.27(0.22–0.31)	68681.02(57655.85–81028.01)	15.89(13.34–18.75)
Eastern Sub-Saharan Africa	245.47(187.47–315.67)	0.34(0.26–0.44)	865.86(663.39–1111.78)	1.22(0.94–1.57)	189.23(143.78–247.30)	0.26(0.20–0.35)	11192.31(8510.25–14582.06)	15.87(12.07–20.68)
High-income Asia Pacific	271.23(229.68–318.50)	0.40(0.34–0.47)	1768.98(1469.57–2122.83)	2.61(2.17–3.14)	60.70(54.79–67.08)	0.08(0.08–0.10)	3652.73(3304.40–4044.39)	5.40(4.89–5.98)
Southern Latin America	19.38(16.81–22.61)	0.10(0.08–0.11)	91.42(76.71–109.80)	0.47(0.40–0.57)	11.19(9.88–12.67)	0.05(0.05–0.06)	677.57(600.44–764.86)	3.55(3.14–4.01)
Southern Sub-Saharan Africa	32.76(30.15–35.52)	0.14(0.13–0.16)	124.76(114.36–136.41)	0.56(0.52–0.62)	24.48(22.45–26.68)	0.11(0.10–0.12)	1463.72(1339.91–1596.93)	6.67(6.11–7.28)
Andean Latin America	14.63(10.86–19.14)	0.05(0.04–0.07)	74.82(53.05–101.11)	0.29(0.20–0.39)	7.35(5.60–9.46)	0.02(0.02–0.03)	447.47(341.07–579.41)	1.74(1.32–2.25)
Australasia	35.35(25.18–48.30)	0.36(0.25–0.49)	234.01(165.37–322.24)	2.41(1.70–3.31)	6.46(5.27–7.85)	0.06(0.05–0.08)	384.04(313.76–464.36)	3.95(3.22–4.77)
Caribbean	35.57(27.33–45.33)	0.19(0.15–0.25)	182.79(140.15–237.60)	1.01(0.77–1.31)	17.77(13.13–22.67)	0.09(0.07–0.12)	1081.50(800.66–1374.90)	5.96(4.41–7.58)
Central Asia	86.32(73.42–100.31)	0.22(0.19–0.26)	402.53(337.01–484.49)	1.06(0.88–1.27)	51.04(44.16–58.44)	0.13(0.11–0.15)	3106.21(2690.02–3538.10)	8.19(7.10–9.33)
Central Europe	108.50(90.75–130.48)	0.30(0.25–0.36)	682.08(560.86–834.33)	1.91(1.57–2.34)	29.88(25.87–35.24)	0.08(0.07–0.09)	1763.98(1522.45–2071.16)	4.95(4.27–5.81)
Central Latin America	94.24(77.01–114.98)	0.09(0.07–0.11)	529.95(425.69–663.37)	0.52(0.42–0.65)	38.16(31.97–45.42)	0.03(0.03–0.04)	2332.76(1953.37–2778.51)	2.31(1.93–2.75)
Central Sub-Saharan Africa	48.37(36.47–62.27)	0.09(0.07–0.12)	179.21(134.14–233.48)	0.34(0.25–0.45)	36.85(27.59–47.52)	0.07(0.05–0.09)	2225.14(1669.15–2878.43)	4.29(3.22–5.55)
East Asia	19959.70(16821.48–23495.57)	3.87(3.26–4.55)	142394.83(119722.43–168255.84)	27.61(23.21–32.62)	2017.41(1721.26–2352.54)	0.39(0.33–0.45)	124496.23(107585.83–144222.44)	24.14(20.86–27.96)
Eastern Europe	221.99(190.88–258.70)	0.32(0.27–0.37)	1366.93(1160.80–1608.46)	1.99(1.69–2.34)	62.42(54.99–71.32)	0.09(0.08–0.10)	3664.80(3243.68–4174.21)	5.33(4.72–6.08)
Eastern Sub-Saharan Africa	469.38(348.95–590.69)	0.28(0.21–0.35)	1732.02(1291.87–2181.96)	1.03(0.77–1.30)	364.76(269.38–462.73)	0.21(0.16–0.27)	21460.22(15867.32–27158.38)	12.86(9.51–16.28)
Global	28562.05(25298.62–32338.40)	0.96(0.85–1.08)	187340.79(164543.64–213665.69)	6.31(5.54–7.19)	6079.20(5600.22–6654.45)	0.20(0.18–0.22)	362910.98(334463.42–394476.78)	12.22(11.26–13.29)
High SDI	2103.82(1839.48–2432.64)	0.63(0.55–0.73)	14780.71(12912.58–17125.12)	4.46(3.89–5.16)	246.58(221.18–277.18)	0.07(0.06–0.08)	15108.22(13587.65–17005.92)	4.56(4.10–5.13)
High-income Asia Pacific	325.51(273.85–381.91)	0.61(0.52–0.72)	2329.24(1958.53–2735.46)	4.43(3.72–5.20)	30.42(26.99–34.67)	0.05(0.05–0.06)	1923.69(1702.39–2206.93)	3.66(3.24–4.20)
High-income North America	402.39(342.53–466.25)	0.33(0.28–0.38)	2815.00(2380.05–3265.21)	2.31(1.95–2.68)	50.27(46.00–54.42)	0.04(0.03–0.04)	3143.84(2875.82–3397.73)	2.58(2.36–2.79)
High-middle SDI	12337.24(10239.63–14691.58)	2.38(1.98–2.84)	87432.32(72247.52–104441.17)	16.92(13.98–20.21)	1389.03(1198.93–1604.49)	0.26(0.23–0.31)	85299.89(74255.87–97409.19)	16.50(14.37–18.85)
Low SDI	984.90(851.82–1124.29)	0.21(0.19–0.25)	3650.40(3152.53–4178.47)	0.81(0.70–0.93)	757.15(655.90–869.47)	0.16(0.14–0.19)	44486.08(38382.41–51004.17)	9.93(8.57–11.39)
Low-middle SDI	2467.42(2204.19–2758.03)	0.33(0.29–0.37)	10828.18(9592.11–12287.67)	1.47(1.30–1.67)	1580.03(1414.99–1753.78)	0.21(0.19–0.23)	92124.13(82562.75–102154.31)	12.52(11.22–13.89)
Middle SDI	10662.02(9145.28–12375.92)	1.14(0.97–1.32)	70613.61(60184.10–82977.34)	7.55(6.43–8.87)	2103.44(1894.85–2341.76)	0.22(0.20–0.25)	125718.75(113190.02–139720.45)	13.44(12.10–14.94)
North Africa and Middle East	1470.60(1213.48–1812.92)	0.56(0.46–0.70)	9502.77(7809.28–11792.41)	3.67(3.01–4.55)	318.00(265.05–382.80)	0.12(0.10–0.14)	19134.87(15931.29–22793.31)	7.39(6.16–8.81)
Oceania	11.44(7.17–17.14)	0.21(0.13–0.31)	42.1603950111687(27.0522521974516–62.3138294385067)	0.77(0.49–1.14)	8.81(5.40–13.36)	0.16(0.09–0.24)	502.80(310.78–755.16)	9.23(5.71–13.87)
South Asia	2352.71(2073.17–2659.64)	0.30(0.26–0.34)	9097.56(7983.91–10299.99)	1.18(1.03–1.33)	1745.35(1535.42–1968.46)	0.22(0.19–0.25)	101195.97(89340.57–113840.23)	13.16(11.61–14.80)
Southeast Asia	1868.10(1551.31–2229.36)	0.68(0.571–0.82)	9394.61(7553.89–11448.59)	3.45(2.78–4.21)	960.77(826.78–1113.10)	0.35(0.30–0.40)	55974.47(48238.18–64617.55)	20.60(17.76–23.79)
Southern Latin America	30.96(21.69–43.61)	0.12(0.08–0.17)	188.64(128.34–269.77)	0.74(0.50–1.06)	9.72(8.13–11.69)	0.03(0.03–0.04)	599.03(501.07–725.23)	2.35(1.97–2.85)
Southern Sub-Saharan Africa	40.23(32.65–48.95)	0.11(0.09–0.14)	157.59(127.70–191.71)	0.46(0.37–0.56)	28.95(23.84–35.04)	0.08(0.07–0.10)	1705.77(1407.36–2059.85)	5.06(4.17–6.11)
Tropical Latin America	126.50(115.14–140.65)	0.14(0.12–0.15)	665.59(592.54–755.80)	0.74(0.66–0.84)	59.89(55.39–64.78)	0.06(0.06–0.07)	3654.81(3382.22–3955.57)	4.10(3.79–4.43)
Western Europe	635.61(546.05–737.68)	0.48(0.41–0.56)	4544.98(3908.82–5266.13)	3.46(2.98–4.01)	60.42(55.88–65.06)	0.04(0.04–0.05)	3846.61(3540.52–4174.56)	2.93(2.70–3.18)
Western Sub-Saharan Africa	223.85(168.65–289.68)	0.12(0.09–0.16)	823.40(616.84–1074.84)	0.45(0.34–0.59)	174.40(128.64–226.26)	0.09(0.07–0.12)	10266.68(7586.82–13306.23)	5.73(4.23–7.42)
1990–2019								
AAPC(95%CI)								
Location	Incidence rate	Prevalence rate	Deaths rate	DALYs rate				
Andean Latin America	0.47 (0.37 to 0.56)	1.34 (1.06 to 1.61)	-0.87 (-1.32 to -0.41)	-0.86 (-1.29 to -0.42)				
Australasia	-0.45(-0.93 to 0.04)	-0.27 (-0.73 to 0.2)	-1.45 (-1.85 to -1.04)	-1.44 (-1.85 to -1.04)				
Caribbean	1.05 (0.82 to 1.28)	1.27 (0.99 to 1.55)	0.69 (0.5 to 0.88)	0.66 (0.46 to 0.86)				
Central Asia	1.64 (1.31 to 1.97)	2.09 (1.79 to 2.38)	1.14 (0.72 to 1.56)	1.11 (0.64 to 1.58)				
Central Europe	2.3 (2.03 to 2.57)	3.32 (3.18 to 3.45)	-0.37 (-0.56 to -0.18)	-0.31 (-0.5 to -0.13)				
Central Latin America	1.45 (1.29 to 1.61)	2.42 (2.03 to 2.8)	-0.3 (-0.45 to -0.15)	-0.28 (-0.43 to -0.13)				
Central Sub-Saharan Africa	-0.8 (-0.88 to -0.73)	-0.72 (-0.8 to -0.65)	-0.75 (-0.88 to -0.61)	-0.8 (-0.94 to -0.65)				
East Asia	3.98 (2.76 to 5.22)	5.46 (4.21 to 6.73)	-2.04 (-2.3 to -1.78)	-1.87 (-2.15 to -1.59)				
Eastern Europe	2.21 (1.13 to 3.3)	2.81 (1.86 to 3.77)	0.58 (-0.58 to 1.75)	0.55 (-0.64 to 1.75)				
Eastern Sub-Saharan Africa	-0.76 (-0.86 to -0.65)	-0.61 (-0.76 to -0.46)	-0.72 (-0.87 to -0.58)	-0.74 (-0.89 to -0.59)				
Global	1.79 (1.03 to 2.55)	2.97 (2.13 to 3.82)	-1.64 (-1.78 to -1.49)	-1.6 (-1.75 to -1.45)				
High SDI	0.77 (0.49 to 1.05)	1.02 (0.82 to 1.22)	-1.86 (-2.01 to -1.7)	-1.71 (-1.86 to -1.56)				
High-income Asia Pacific	1.4 (0.97 to 1.85)	1.74 (1.13 to 2.36)	-1.69 (-2.05 to -1.32)	-1.49 (-1.76 to -1.23)				
High-income North America	0.02 (-0.21 to 0.24)	0.19 (-0.05 to 0.42)	-1.38 (-1.66 to -1.11)	-1.3 (-1.57 to -1.03)				
High-middle SDI	4 (3.41 to 4.58)	5.28 (4.5 to 6.07)	-1.19 (-1.38 to -1.01)	-1.05 (-1.28 to -0.81)				
Low SDI	-0.79 (-0.99 to -0.59)	-0.6 (-0.77 to -0.43)	-0.88 (-1.08 to -0.68)	-0.89 (-1.1 to -0.68)				
Low-middle SDI	-0.24 (-0.46 to -0.02)	0.35 (0.11 to 0.59)	-0.88 (-1.25 to -0.5)	-0.93 (-1.29 to -0.56)				
Middle SDI	1.84 (1.36 to 2.33)	3.54 (2.84 to 4.25)	-2.27 (-2.44 to -2.09)	-2.25 (-2.44 to -2.06)				
North Africa and Middle East	1.31 (1.11 to 1.51)	2.21 (2.13 to 2.29)	-1.79 (-1.97 to -1.62)	-1.8 (-1.96 to -1.63)				
Oceania	0.19 (0.04 to 0.33)	0.21 (0.06 to 0.35)	0.19 (0.08 to 0.29)	0.17 (0.06 to 0.28)				
South Asia	-0.43 (-0.9 to 0.04)	-0.18 (-0.55 to 0.2)	-0.76 (-1.47 to -0.05)	-0.74 (-1.21 to -0.26)				
Southeast Asia	0.38 (0.28 to 0.47)	1.28 (1.16 to 1.4)	-0.86 (-0.95 to -0.76)	-0.9 (-1.01 to -0.79)				
Southern Latin America	0.62 (0.39 to 0.84)	1.52 (1.29 to 1.76)	-1.48 (-1.89 to -1.06)	-1.42 (-1.83 to -1.01)				
Southern Sub-Saharan Africa	-0.9 (-1.7 to -0.09)	-0.8 (-1.58 to -0.01)	-0.97 (-1.87 to -0.07)	-1.06 (-1.75 to -0.37)				
Tropical Latin America	2.14 (1.87 to 2.41)	2.96 (2.68 to 3.24)	0.85 (0.61 to 1.08)	0.88 (0.65 to 1.11)				
Western Europe	-0.04 (-0.3 to 0.21)	0.15 (-0.12 to 0.42)	-2.16 (-2.31 to -2.01)	-2.03 (-2.18 to -1.88)				
Western Sub-Saharan Africa	0.33 (0.17 to 0.5)	0.46 (0.25 to 0.68)	0.25 (0.08 to 0.41)	0.28 (0.1 to 0.45)				

### Disease burden in the country

**Fig. 1:**
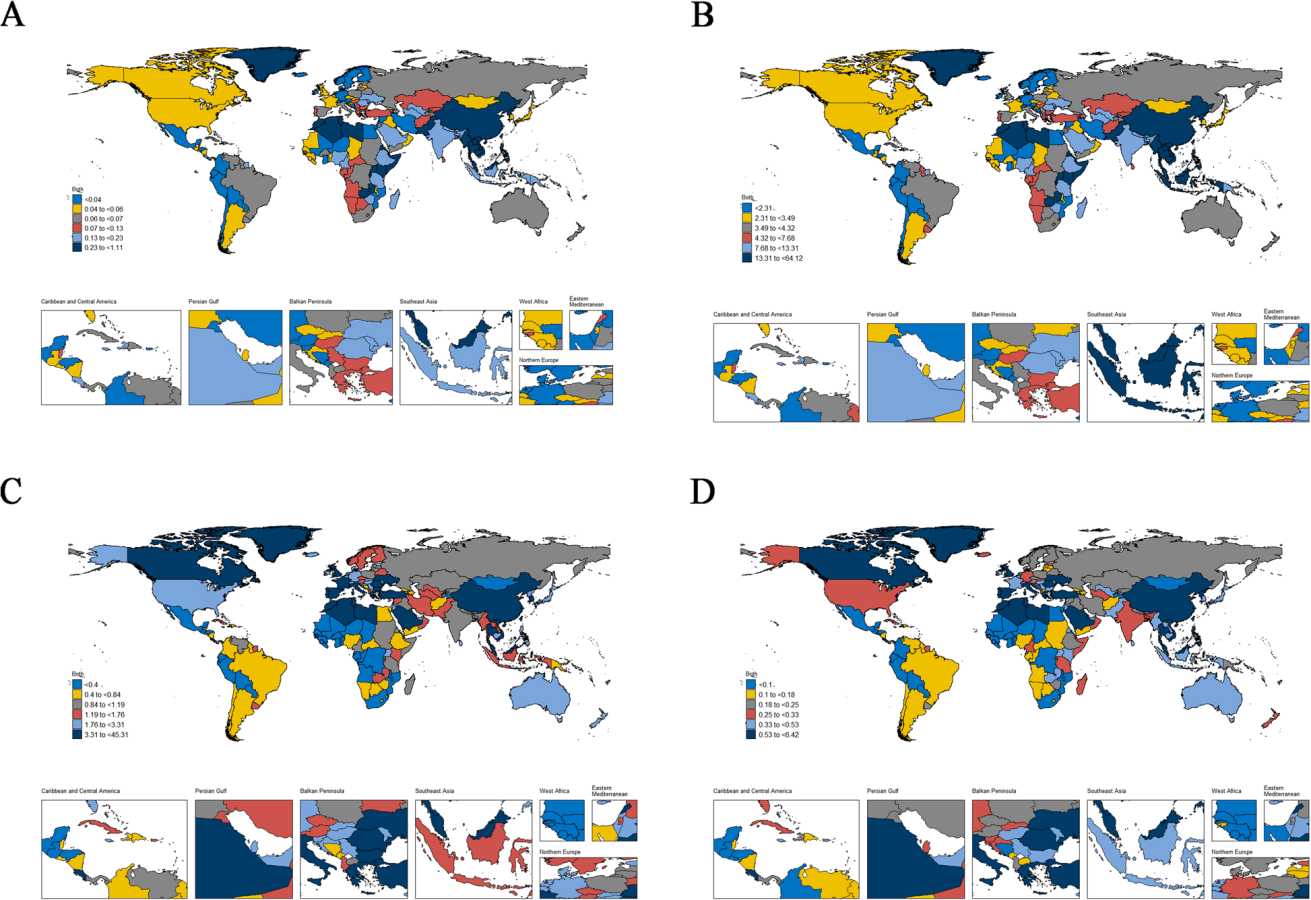
Geographical distribution of Age-standardized Deaths (**A**), Age-standardized DALYs (**B**), Age-standardized Prevalence (**C**) and Age-standardized Incidence (**D**)

In 2019, the countries with the lowest ASDR were part of Central America and Northern Europe countries, such as Honduras, Finland and Norway; the countries with the highest were part of Southeast Asia, Malay Archipelago and Arctic regions countries, such as Malaysia, Brunei Darussalam, Taiwan (China) and Greenland. The countries with the lowest ASIR were part of Central America, West Africa and Southern Africa countries,such as Honduras, Niger, Mozambique and Mali; the countries with the highest ASIR were part of Southeast Asia, East Asia and Northern Africa countries, such as Taiwan (China), Singapore, China, Malaysia and Tunisia. The countries with the lowest ASPR were part of Central America, West Africa and Southern Africa countries, such as Niger, Honduras, Mozambique and Mali; the countries with the highest ASPR were part of Southeast Asia, East Asia and Northern Africa countries, such as Taiwan (China), Singapore, China, Tunisiaand Algeria. The countries with the lowest age-standardized DALYs were part of Central America, Northern Europe and West Africa countries, such as Honduras, Finland, Niger and Norway; the countries with the highest age-standardized DALYs were part of Southeast Asia, Malay Archipelago and Arctic regions countries, such as Malaysia, Brunei Darussalam, Taiwan (China), Greenland and Viet Nam See Fig. 1 for details.

### The incidence, prevalence, deaths, DALYs of NPC at regional level

**Fig. 2:**
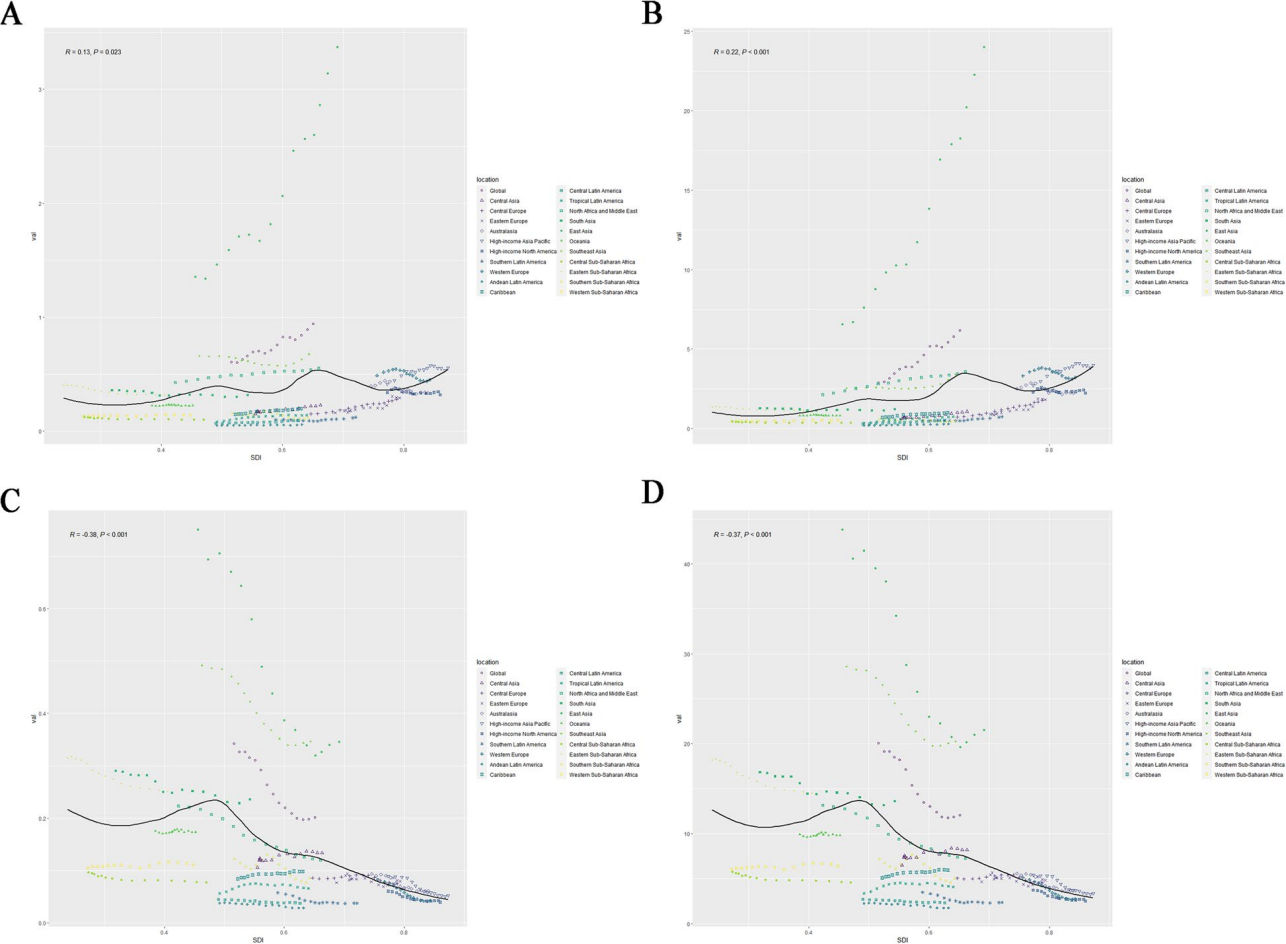
Age-standardized Incidence (**A**), Age-standardized Prevalence (**B**), Age-standardized Deaths (**C**) and Age-standardized DALYs (**D**) for nasopharyngeal carcinoma by SDI, 1990–2019, and expected value-based SDI

Figure 2A shows the Incidence rates smooth curve of DALYs across 21 regions. Using a smoothed spline model, R = 0.13 and *p* < 0.05 determined that the incidence rates have a steady fluctuation trend with increasing SDI. In the majority of regions, the incidence rates remained stable; East Asia showed a increasing trend.

Figure 2B shows the prevalence rates smooth curve of DALYs across 21 regions. Using a smoothed spline model, R = 0.22 and *p* < 0.001 determined that the prevalence rates generally have a increasing trend with increasing SDI. In the majority of regions, the prevalence rates remained stable; East Asia showed a increasing trend.

Figure 2C illustrates the deaths rates smooth curve for 21 regions. The deaths rates generally exhibit a decreasing trend with increasing SDI, as determined by the smoothed spline model, R = -0.38, *p* < 0.001. Four regions (South Asia, East Asia, North Africa and Middle East) exhibited a decreasing incidence trend. In comparison, other regions exhibited a remained stable trend.

Figure 2D shows the trend of DALYs rates in 21 regions. Smoothed spline model demonstrated that the DALYs rates generally exhibit a decreasing trend with increasing SDI; the analysis result was R = -0.37, *p* < 0.001. Four regions (South Asia, East Asia, North Africa and Middle East) exhibited a decreasing incidence trend. In comparison, other regions exhibited a remained stable trend.

### The incidence, prevalence, deaths, DALYs of NPC at country level

**Fig. 3:**
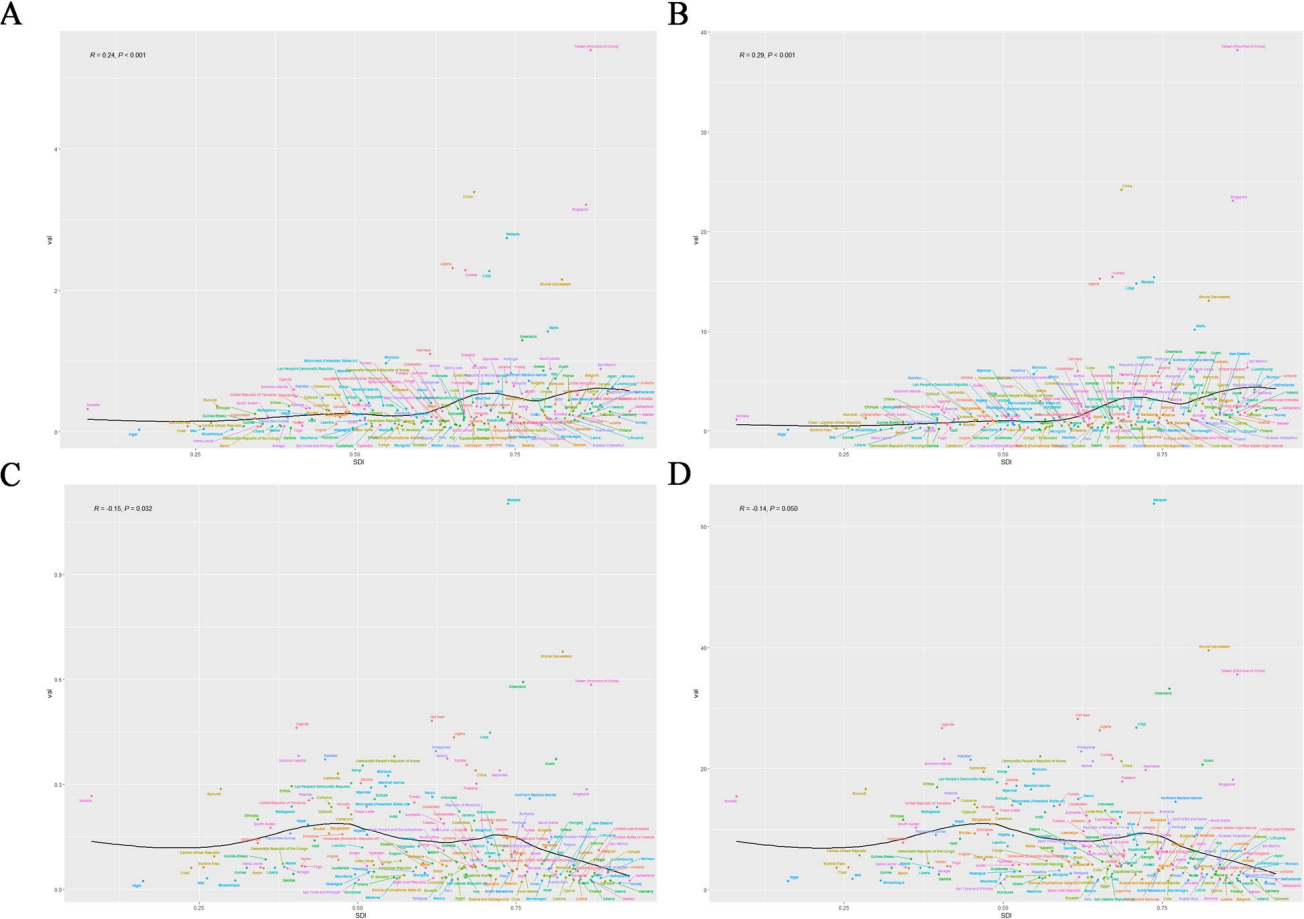
Incidence rate, prevalence rate, Deaths rate and DALYs rate of nasopharyngeal carcinoma by 204 countries and terri-ries and SDI in 2019

Figures 3A show the incidence rates across 204 countries. The incidence of the smoothed spline model increases with increasing SDI. Eight country's (China, Taiwan(China), Algeria, Tunisia, Libya, Malta, Brunel and Greenland) incidence rate is significantly higher the smooth curve.

Figures 3B show the prevalence rates across 204 countries. The prevalence of the smoothed spline model increases with increasing SDI. Seven nations (China, Taiwan(China), Algeria, Tunisia, Libya, Malta, Brunel)had prevalence rate is significantly higher the smooth curve.

Figures 3C show the deaths rates for 204 countries. With increasing SDI, the smoothed spline model demonstrates an stable and decreasing trend in deaths.Brunel,Greenland and Taiwan(China) had prevalence rates significantly higher the smooth curve.

Figures 3D display the DALYs rates for 204 countries. As SDI rises, refined spline models indicate an stable and decreasing DALYs rate trend. However Brunel, Greenland and Taiwan(China) had prevalence rates significantly higher the smooth curve.

### Decomposition analysis of change in DALYs

**Fig. 4:**
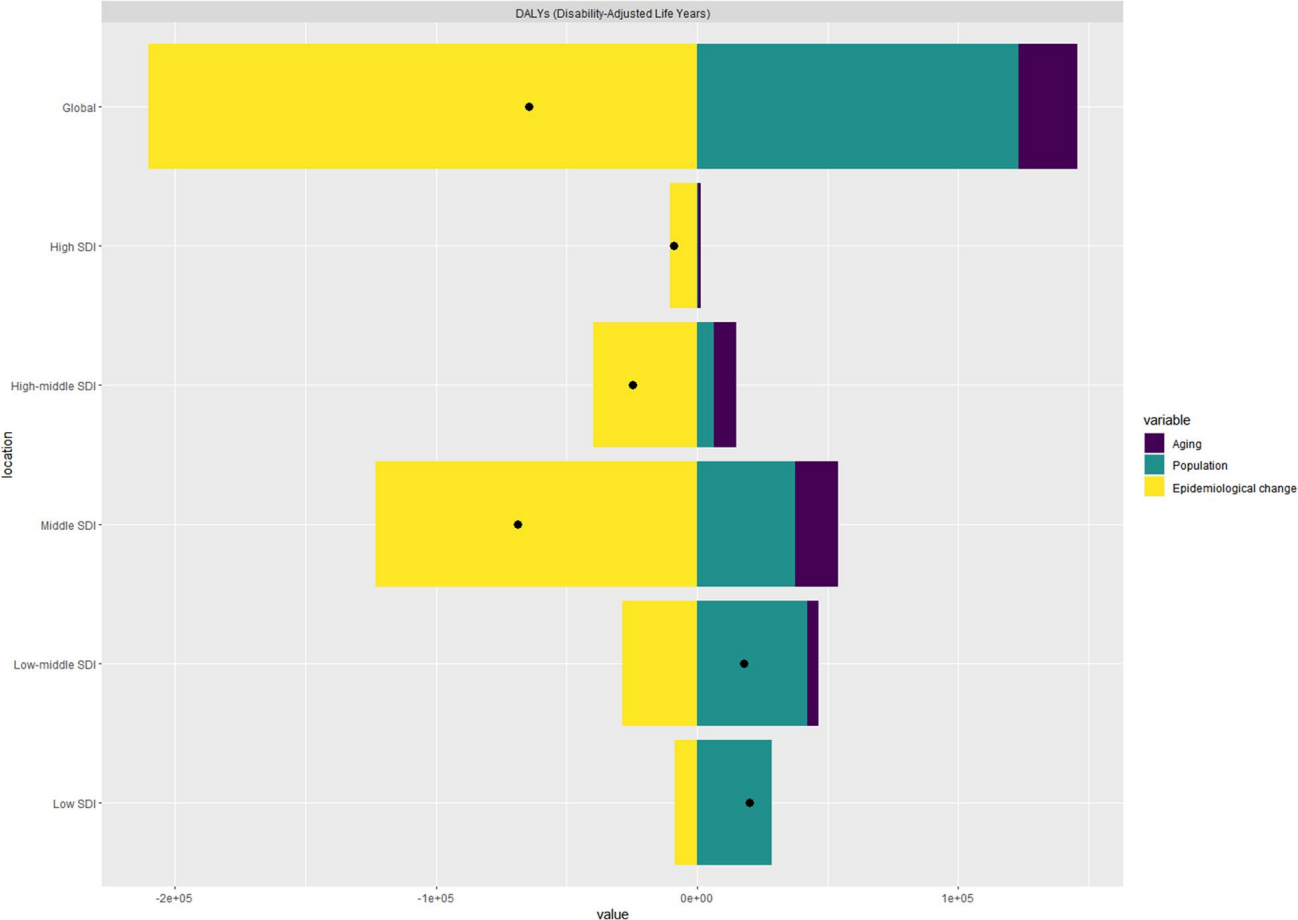
Changes in nasopharyngeal carcinoma disability-adjusted life years (DALYs) according to population-level determinants of population growth, aging, and epidemiological change from 1990 to 2019 at the global level and by Sociodemographic Index (SDI) quintile

Decomposition analysis results for the global level and the SDI regions are shown in Fig. 4. At the global level,-190.75% of the changes in DALYs were attributed to population growth, -34.78% to population aging, and 325.54% to epidemiologic changes. The contributions of population aging in the SDI regions were high SDI (-10.75%), high-mid SDI (-34.25%), middle SDI (-24.14%), low-mid SDI (23.98%), and low SDI (0.11%). The contributions of population growth in the SDI regions were high SDI (-5.49%), high-mid SDI (-26.41%), middle SDI (-54.68%), low-middle SDI (235.94%), and low SDI (143.44%). The contribution of epidemiological changes in the SDI regions was high SDI (116.25%), high-middle SDI (160.67%), middle SDI (178.82%), low-middle SDI (-159.94%), and low SDI (-43.55%), respectively.

### Frontier analysis based on age-standardized DALYs

**Fig. 5:**
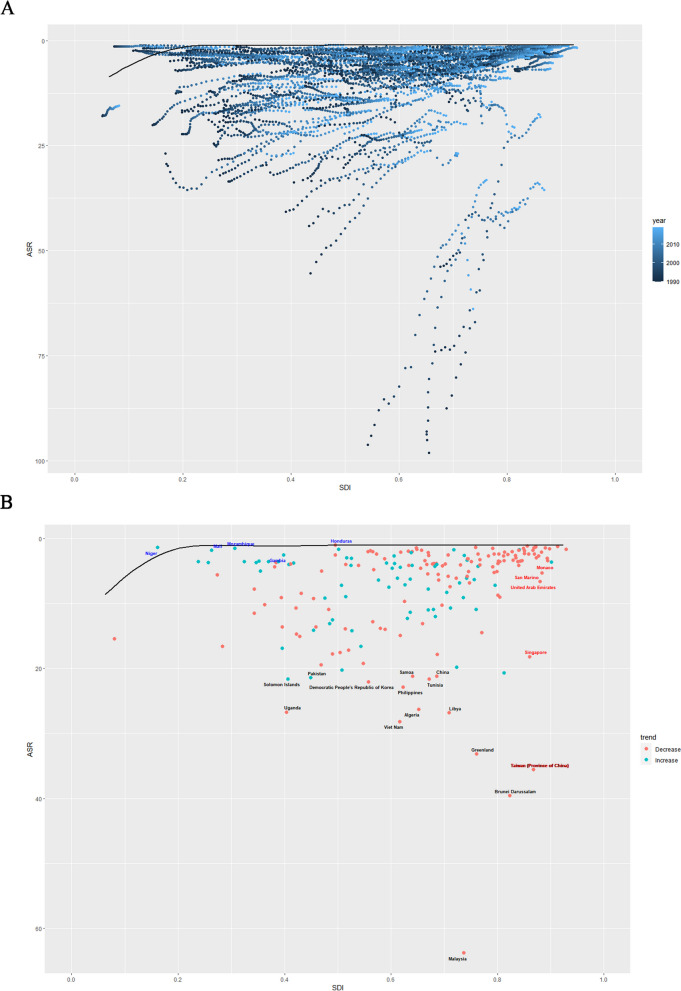
Frontier analysis on the basis of sociodemographic-index and age-standardized DALYs per 100,000 of nasopharyngeal carcinoma from 1990 to 2019. (**A**)1990–2019; (**B**) 2019

Frontier analysis results for 204 countries are shown in Fig. 5. Details of the frontier analysis have previously been disclosed in the literature. The black labels represent countries and regions with the highest effective difference, such as Solomon Islands, Pakistan, Uganda, Samoa, China, Democratic People's Republic of Korea, Philippines, Tunisia, Algeria, Viet Nam, Libya, Greenland Brunei Darussalam, Taiwan(China). Blue labels represent countries and regions with low SDI (< 0.45) and low effective difference, such as Niger, Mall, Mozambique, Gambia, Honduras; Red labels represent countries and regions with high SDI (> 0.85) and relatively significant difference in development levels, such as Monaco, San Marino, United Arab Emirates, Singapore and Taiwan(China).

### Burden of disease projections

**Fig. 6:**
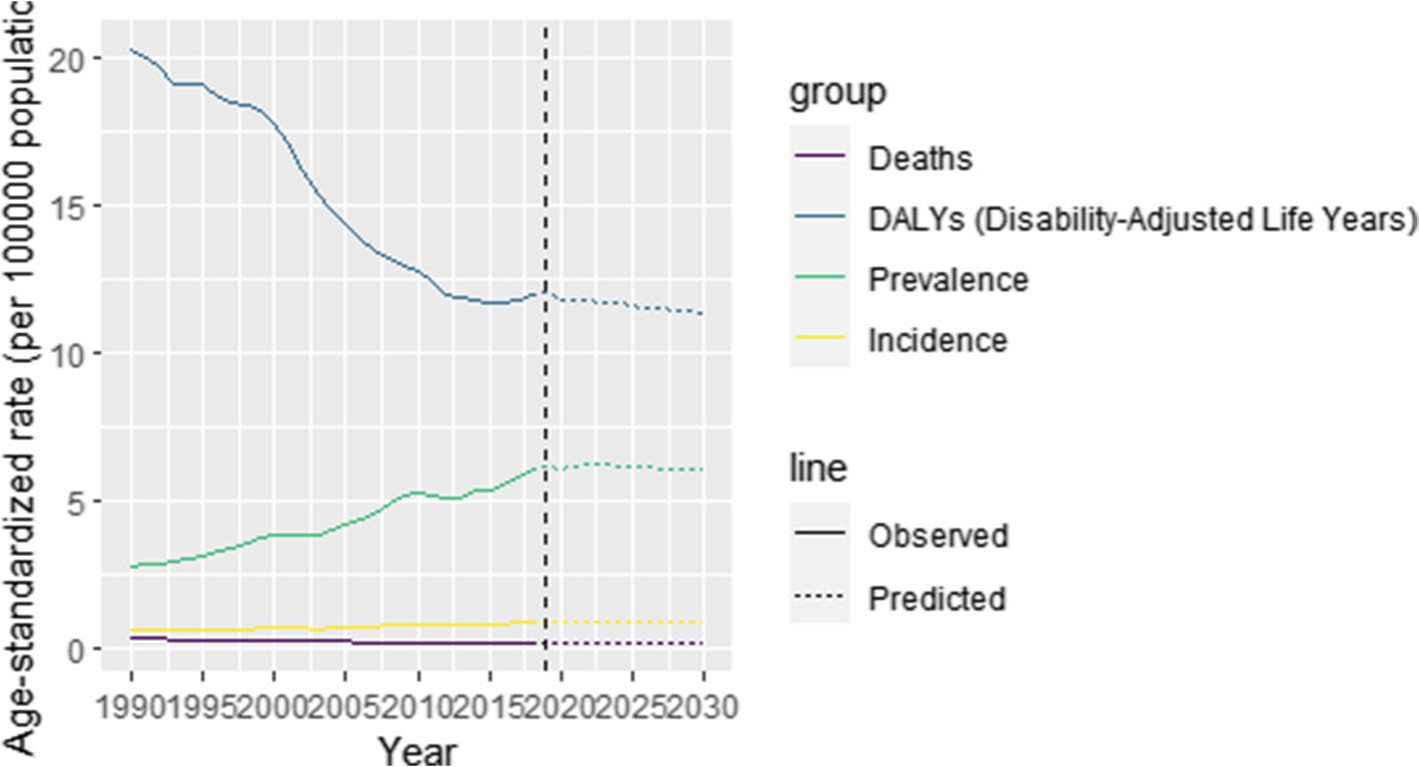
The ASDR, age-standardised DALYs, ASPR and ASIR for global nasopharyngeal cancer for the observational period (1990–2019) and the projection period (2020–2030)

Based on the GBD 2019 study, this study projects the global burden of disease for NPC from 2020 to 2030. The results of the projections are shown in Fig. 6. The global projections show that ASIR, ASPR, and ASDR remain stable and DALYs show a decreasing trend by 2030. By 2030, there will be 28,148 incident cases, 5,888 deaths, 194,172 prevalent cases and 362,937 DALYs. Cases and 329,383 DALYs (Table 2).

**Table 2:** The number of cases of prediction of incidence, prevalence, deaths and DALYs of nasopharyngeal carcinoma from 2020–2030

Location	2020				2030			
	Incidence	Prevalence	Deaths	DALYs	Incidence	Prevalence	Deaths	DALYs
	The number of cases				The number of cases			
Andean Latin America	14	72	7	416	17	88	8	403
Australasia	34	232	6	378	31	222	6	376
Caribbean	36	183	18	1101	38	197	19	1129
Central Asia	87	412	52	3136	99	512	56	3331
Central Europe	111	693	30	1709	112	705	26	1350
Central Latin America	102	578	41	2500	123	721	44	2668
Central Sub-Saharan Africa	48	177	37	2247	61	225	47	2954
East Asia	20295	144704	2044	125405	17682	126613	1602	98178
Eastern Europe	270	1694	62	4003	333	2218	45	3875
Eastern Sub-Saharan Africa	482	1795	377	22503	623	2417	489	30166
Global	28235	187592	6020	362169	28148	194172	5888	362937
High-income Asia Pacific	303	2149	28	1773	230	1621	22	1354
High-income North America	421	2982	52	3349	395	2928	46	3377
North Africa and Middle East	1457	9442	309	18710	1551	10363	282	17598
Oceania	11	42	9	506	14	51	11	608
South Asia	2317	8868	1735	98886	2492	9666	1835	99929
Southeast Asia	1856	9371	946	55161	2225	12108	974	56818
Southern Latin America	30	188	9	590	33	223	9	603
Southern Sub-Saharan Africa	38	148	27	1571	39	158	28	1534
Tropical Latin America	127	652	60	3579	132	655	58	3169
Western Europe	598	4331	55	3625	510	3867	43	3188
Western Sub-Saharan Africa	243	890	190	10971	363	1331	282	15569

## Discussion

To the best of our knowledge, this study is the first to comprehensively analyse the burden of nasopharyngeal cancer in young people and changing trends at the global, regional and national levels, analysing incidence, prevalence and rates of DALYs at the global and regional levels over the last 30 years, and examining the incidence, prevalence and rates of DALYs in different regions and countries as the level of the SDI varies, adding detail to the limited epidemiological data available on NPC in young people. Based on two years of data, 1990 and 2019, overall, the number of deaths and mortality rates among young people with NPC have declined globally, with an AAPC of -1.64 (-1.78 to -1.49), which may be attributed to the growth of the global economy, improved health care services, and improved medical technology. However the incidence of NPC did increase significantly. Compared to 1990, the incidence in 2019 showed a double increase and the prevalence showed a triple increase, with an AAPC of 1.79 (1.03 to 2.55) and 2.97 (2.13 to 3.82). Although the number of deaths of NPC patients declined globally during this 30-year period, the number of patients with the disease increased dramatically. On the one hand, this may be due to the fact that early cases of NPC circa in the 1990were often poorly described, misdiagnosed or misclassified. With improved screening tools, the rate of misdiagnosis has been greatly reduced [11]. On the other hand, the prevalence of risk factors such as dust, alcohol and tobacco use has also had an impact on the number of cases. Fortunately, however, DALYs for NPC in young people are declining globally, suggesting that the burden of NPC on individuals and society is decreasing in this group [12–14].

The SDI is a composite indicator that quantifies the level of socio-demographic development, which is steadily increasing in most countries. The significant association between the SDI and the burden of disease indicator reinforces the impact of socio-demographic differences on the burden of disease. As the SDI increases, we can observe a steady downward trend in mortality and DALYs in most of the 21 regions. Moreover, the declining trend is more pronounced in four regions, namely South Asia, East Asia, North Africa and Middle East, which may be attributed to the fact that with the continuous socio-economic development, developing countries have improved the prognosis of patients with NPC due to the improvement of treatment technology and awareness of the disease. However, in this general environment, some countries still have high mortality rates and DALYs, such as Brunel, Greenland and Taiwan (China), which also have the highest ASDR and age-standardised DALYs, Viet Nam also had high age-standardised DALYs. This suggests that prevention and treatment of nasopharyngeal cancer in these countries are problematic and need to be strengthened. To further investigate the impact of different factors on NPC DALYs over the past 30 years, we decomposed the raw DALYs by three dimensions: population, ageing and epidemiological changes. Overall, global NPC DALYs decreased significantly. This reduction was primarily driven by age-standardised mortality and morbidity, or epidemiological change, which accounted for 325.54% of the reduction in the burden of NPC DALYs. However, changes in NPC bur/den varied by level of development. Dividing the SDI into five levels, in high, medium–high and medium SDI regions, the main factor affecting DALYs remained epidemiological changes, with high, medium–high and medium SDI regions contributing majorly to the decline in the global burden of NPC DALYs, accounting for 116.25%, 160.67% and 178.82% of the reduction in the burden of NPC DALYs. However, in the medium–low and low SDI regions, the main factor affecting DALYs becomes population growth, which has a neutralising effect on the decline of DALYs, accounting for 235.94% and 143.44% of the increase in the burden of NPC DALYs, respectively, and similarly, population ageing, which has a relatively small impact on the global NPC DALYs.

To better understand the potential improvements in NPC DALYs rates that might be achieved given a country's development status, we built a frontier analysis based on age-standardised DALY rates and SDI using data from 1990 to 2019. The frontier lines depict the countries and regions with the lowest DALYs at the corresponding SDI. The distance from the frontier is referred to as the effective difference and represents the gap between a country's observed and potentially achievable DALYs; this gap can be reduced or eliminated depending on the socio-demographic resources of the country or territory. The effective difference from the border was calculated for each country and territory using the 2019 DALYs and SDI. East Asia, Southeast Asia, North Africa and some island countries in the Pacific Ocean have the highest effective variance. Part of Central America, West Africa and Southern Africa countries, despite having low SDI, also have the lowest DALYs and show the best DALYs in resource-limited settings, and they can serve as models for optimising health outcomes in resource-poor settings. In contrast, some high SDI countries and regions lagged behind, such as part of East Asia, Southeast Asia, Europe and the Middle East countries. This observation suggests that future work should identify the drivers of success in exemplary countries and the potential causes of countries with high DALYs, and that addressing this gap may help inform efforts to reduce the burden of NPC.

With the increase of SDI, the incidence and prevalence rates in most of the 21 regions showed a steady increase in general, especially in East Asia, which showed a significant increase, including China and Taiwan (China), both of which also had the highest ASIR and ASPR. This cancer is very common in provinces in the eastern to south-eastern regions of China, such as Guangdong, Guangxi, Zhongshan and Hong Kong [15]. It is noteworthy that the incidence and prevalence of NPC in Taiwan of China is high, and the mortality rate is also high. This may be due to the fact that the cultivation and consumption of betel nut is very common in Taiwan, and under the influence of alcohol consumption and smoking, the incidence of head and neck cancers remains high in the region [16]. Moreover, the residents of this coastal area are fond of eating salted and dried fish, which contains high levels of nitrites and their precursors, which are highly carcinogenic, and EBV activators, which can promote NPC [17–19]. This suggests that the relevant health authorities in Taiwan of China need to pay more attention to these high risk factors and develop some targeted preventive measures, otherwise it will be difficult to adapt to the upcoming challenges posed by the burden of NPC. In other regions, there are also countries that show outstanding incidence and prevalence rates such as Brunel, Algeria, Tunisia, Libya, Malta, where Tunisia have relatively the highest ASIR and ASPR. ASPR are also relatively highest. On the one hand, this may be due to the fact that the risk of NPC is closely related to the degree of social mixing, as some people from high incidence areas migrate to these areas and intermarry with the local population [20]. On the other hand, it may be that the genetic susceptibility of the local indigenous population also has an impact on the incidence of NPC [21]. In addition, the uneven development of health care across regions and countries is also important for regions and countries with high mortality rates and high DAYS to have equitable access to health care and living conditions.

The current situation poses a serious challenge to public health systems in South and South-East Asia and parts of Africa and Europe, in particular, where rapid growth, if left unchecked, could lead to a "nasopharyngeal cancer epidemic" in these regions in the near future. Therefore, effective measures should be taken to curb this situation, such as improving the specificity of testing, reducing overdiagnosis, promoting individualised treatment and developing healthier lifestyles. By understanding the trends and distribution of the NPC burden in different countries and regions, we can better explore the underlying factors affecting the NPC burden, and also suggest policy makers to rationally allocate resources for more targeted prevention.

Based on the GBD 2019 study, this study predicts the global burden of disease of NPC from 2020 to 2030. According to the prediction, the ASIR, ASPR, and ASDR of NPC will remain unchanged in the future, but the DALYs will show a decreasing trend, which suggests that the impact of NPC on the burden of young people will continue to decrease in the future but the number of diseases and deaths will continue to increase. Therefore, health issues related to NPC must be better managed, and this will remain a global public health issue that requires the cooperation of all in an effort to eliminate the burden of disease caused by NPC by controlling the high risk factors and improving healthcare, prevention, and promotion.

Our study also has some limitations. Firstly, the data are from GBD 2019, which makes it difficult to avoid inaccuracies entirely, as some values are estimates rather than direct measurements. Given the current state of poor disease surveillance in many countries, GBD 2019 would be a more systematic study to provide a perspective on the burden of NPC. Secondly, due to the lack of data, we were unable to analyse the relationship between stage/grading at cancer diagnosis and mortality-morbidity; furthermore, we were unable to adjust for the impact of screening practices in the association between SDI and NPC burden. After addressing these limitations, future more comprehensive population-based epidemiological studies are needed to confirm our results. Shortcomings of this study: although the data were obtained from the GBD database, and the GBD methodology and results are considered state-of-the-art, robust, and reliable, there is still a need is data inaccuracies. In underdeveloped countries, cancer detection systems are weak, which may lead to under-registration of cancer. Secondly, there are differences in medical technology in different countries, which may lead to misdiagnosis and underdiagnosis of NPC diagnosis.

## Conclusions

NPC incidence in young people is a major global problem. Mortality and DALYs have been decreasing in the last 30 years, but incidence and prevalence have been increasing, especially in East Asia. Moreover, there are large differences in incidence, prevalence/mortality and DALYs among different regions and countries. Among them, China, Taiwan and some Southeast Asian countries not only have high incidence and prevalence rates, but also still have high mortality rates and DALYs. This is particularly important for these countries to pay attention to the health problems related to NPC, and the projected burden of disease from 2020 to 2030 shows that ASIR, ASPR, and ASDR remain unchanged, but DALYs show a decreasing trend. Therefore, there is still a need to focus on the incidence of NPC in young people and to target interventions to improve the current situation.

## Data Availability

The data used are publicly available online on the website of the Institute for Health Metrics and Evaluation (IHME) (http://ghdx.healthdata.org/gbd-resultstool).
